# Tertiary Cytoreduction for Isolated Lymphnode Recurrence (ILNR) Ovarian Cancer in a BRCA2 Mutated Patient: Our Experience and Prevalence of BRCA 1 or 2 Genes Mutational Status in ILNR

**DOI:** 10.3390/medicina59030606

**Published:** 2023-03-19

**Authors:** Matteo Bruno, Manuela Ludovisi, Carlo Ronsini, Giulia Capanna, Guglielmo Stabile, Maurizio Guido

**Affiliations:** 1 Obstetrics and Gynaecology Unit, San Salvatore Hospital, 67100 L’Aquila, Italy; 2 Department of Life, Health and Environmental Sciences, University of L’Aquila, 67100 L’Aquila, Italy; 3 Department of Woman, Child and General and Specialized Surgery, Obstetrics and Gynecology Unit, University of Campania “Luigi Vanvitelli”, 80138 Naples, Italy; 4 Institute for Maternal and Child Health-IRCCS “Burlo Garofolo”, Department of Obstetrics and Gynaecology, 34137 Trieste, Italy

**Keywords:** tertiary cytoreductive surgery, isolated lymphnode recurrence, BRCA mutated patient

## Abstract

We report the case of a tertiary cytoreductive surgery for isolated lymph-node recurrence (ILNR) in a 54-years old Brest cancer 2 (BRCA 2) mutated patients, with a personal history of ovarian cancer previously treated elsewhere. She was admitted to our department for a suspected isolated lymph-nodal pelvic recurrence. A positron emission tomography acquisition with contrast enhanced computed tomography (PET-CT) scan revealed an increased node at the level of the right external iliac (SUV 6.9) in correspondence with the obturator nerve, which was confirmed by transvaginal ultrasound. Since the recurrence was in a single site and the patient had previously undergone three lines of chemotherapy and maintenance with Poly(ADP-ribose) polymerase (PARP) inhibitors, we decided to perform tertiary cytoreductive surgery by minimally invasive laparoscopic approach. After gradual and careful isolation of the obturator nerve, lumbo-sacral trunk and venous vessels afferent to the external and internal iliac vein, the suspected node has been removed. No intra- and postoperative complications occurred. The patient was discharged three days after procedure. We decided to quarterly follow-up; actually, after 16 months no recurrence was detected. Several studies have reported ILNR as a unique clinical disease with low growth rate and less chemosensitivity; this can lead to considered ILNR more susceptible to take advantage of surgical treatment, even in case of second or third recurrence. The BRCA mutational status seems to play a role in the decision-making process in the approach to patients with platinum sensitive relapse of ovarian cancer or in specific isolated forms of recurrence such as the hepatic one. However, data on frequency and prognostic impact of BRCA gene mutation in ILNR are very limited. In this article we investigated the role of BRCA 1 or 2 mutational status in this rare pattern of recurrence according to more recent advances in literature.

## 1. Introduction

The major clinical challenge in epithelial ovarian cancer (EOC) is the management of relapse, which occurs in around 60–75% of patients within two years from diagnosis [1,2]. Currently, the goals of treatment of recurrent ovarian cancer (ROC) are mainly focused on increasing progression free-survival (PFS) of these patients and optimizing their quality of life. In the last years, status of *BRCA* genes or homologous recombination deficiency (HRD), and the pattern of relapse have played an increasing role in suggesting potential appropriate personalized treatment [3,4,5]. The introduction of PARP-inhibitors (PARPi) as complementary therapies has reduced the relapses and improved the interval free from disease of the patients. By the way, secondary surgical cytoreduction is recognized as one of the tools to improve survival outcomes when the recurrence occurs. The role of secondary cytoreduction has been evaluated in three randomized trials: Gynecologic Oncology Group 213 (GOG 213), DEKSTOP III/ENGOT-ov 20 and SOC-1 [6,7,8]. However, the majority of patients will as well develop a secondary relapse. In those cases the non-surgical management is usually preferred in lack of evidences for the best treatment. A recent meta-analysis has demonstrated that optimal tertiary cytoreduction surgery with an absence of residual tumor was associated with improved overall survival (OS) and PFS compared to suboptimal tertiary cytoreductive surgery [9], and this is in line with previous retrospective analysis of tertiary cytoreduction [10,11]. Such results are true when applied to a highly selected population (single recurrence, platinum-free interval >12 months) that could benefit from tertiary surgery. Lymph node involvement occurs in up to 30% of EOC relapses; however, isolated lymph node recurrence (ILNR) in EOC is rare, and studies have been limited [12,13,14,15]. ILNRs could derive from an initial refuge for microscopic disease [16], accounting for 1–5% of EOC cases, principally localized in the para-aortic lymphnodes [17]. Several studies have described ILNR as a unique clinical disease with a low growth rate and less chemosensitivity [18,19,20]. These observations corroborate the role of surgery in managing the ILNR, even in case of second or third recurrence. On the other hand, assessment of *BRCA* gene mutation could help to predict the platinum sensitive disease [5,21]. However, we need more data about the correlation between mutational *BRCA* status and ILNR [17,22].

In this article, we report our experience with a laparoscopic eradication of pelvic ILNR in a *BRCA 2* mutated patient previously treated by PARPi. We also systematically investigated the role of *BRCA* 1 *or* 2 mutational status in this rare pattern of recurrence according to more recent advances in literature.

## 2. Case Report

We present the case of a 54-years old woman, with a personal history of ovarian cancer previously treated in another hospital. In 2016, because of ascites and carcinomatosis, she underwent to radical hysterectomy with bilateral salpingo-oophorectomy and rectosigmoid resection with primary anastomosis, radical omentectomy, performed by a midline laparotomy, with complete cytoreduction (CC-0). Histological examination revealed a high grade serous ovarian cancer (HGSOC), FIGO stage IIIC. Six cycles of Paclitaxel 175 mg/m^2^ and Carboplatin AUC 6 every 21 days were administered.

**Figure 1:**
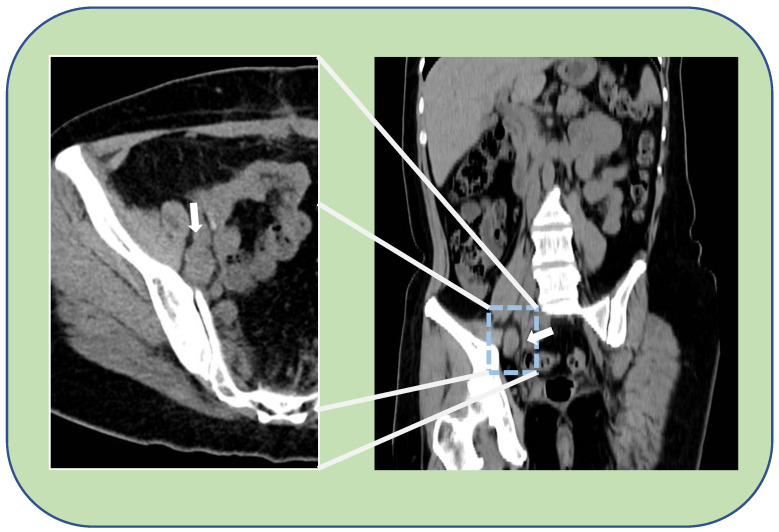
Thick white arrow shows an enlarged, pathologic, lymphnode of about 2 cm in the right obturator region. On the left, a detail of the right half-pelvic cavity on the axial view. On the right, a coronal view of the CT-PET performed during the follow-up.

**Figure 2:**
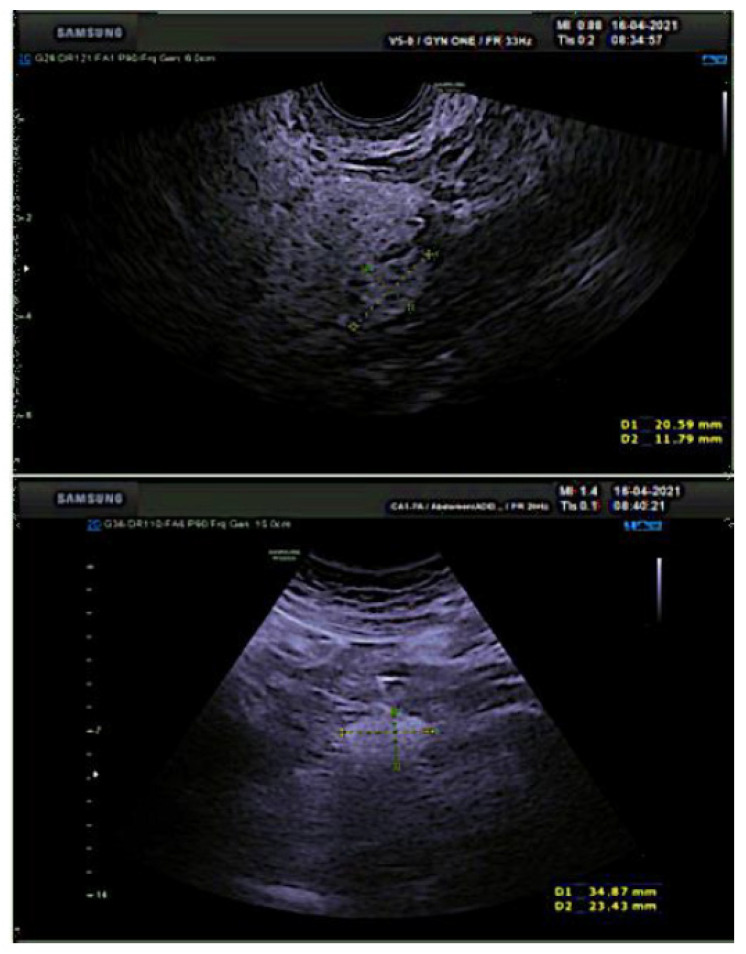
Ultrasound preoperative image showing isolated lymph node recurrence (ILNR) 20 × 34 × 23 mm in size, attached to the external iliac vessels.

After 12 months of platinum-free interval an isolated pelvic peritoneal recurrence occurred. Additional six cycles of platinum-based chemotherapy were administered achieving complete disease remission. She was successively diagnosed with a germ-line *BRCA* 2 mutation. A further recurrence occurred 15 months after the end of second-line chemotherapy. For this, she underwent to surgical management with pararectal node excision, hepatic node excision and diaphragm nodes ablation by laparotomy, with an optimal residual tumor (CC-1). Three cycles of platinum-based chemotherapy followed the surgery, and Olaparib was administrated until it was discontinued, 24 months later, following an episode of transient subocclusion, managed with nonsurgical treatment. After further 22 months, a PET-CT scan revealed FDG uptake (SUV 6.9) at a right external iliac increased node (24 mm in maximum diameter) in correspondence with the obturator nerve (Figure 1). The trans-obturator recurrence was also confirmed by a transvaginal ultrasound (TV-US) performed by an ultrasonographer with a high expertise in the field of oncological gynecology (Figure 2). She was thus scheduled for surgery at our Center in consideration of the absence of blood vessels invasion, the single site of recurrence and previous therapy with PARPi.

**Figure 3:**
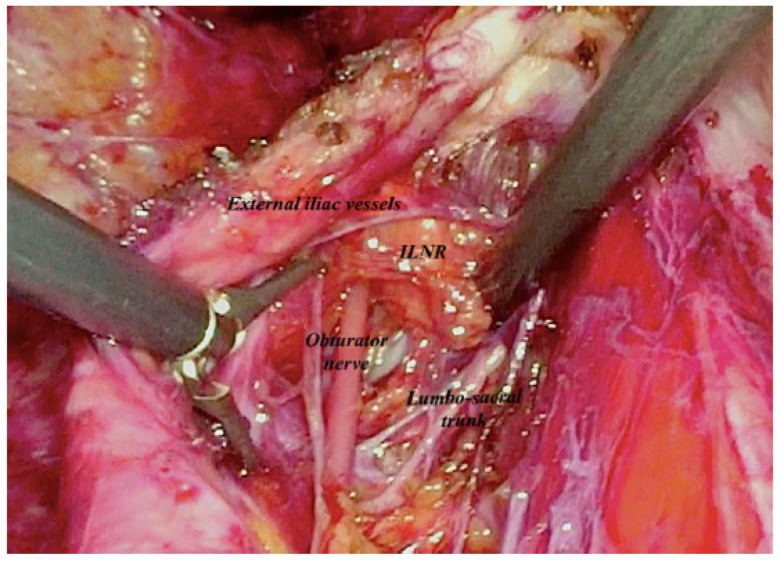
Laparoscopic intraoperative image showing access to ILNR by the ileo-lumbar space.

**Figure 4:**
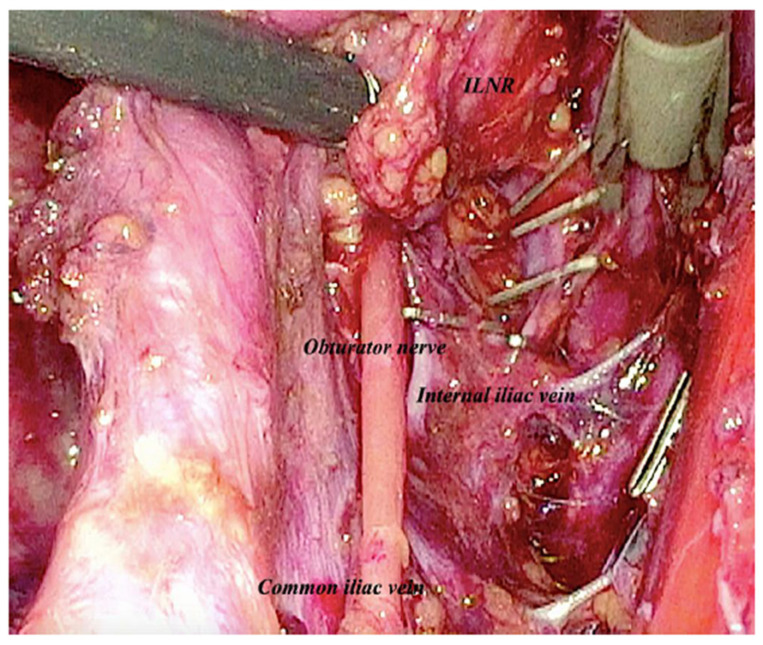
Laparoscopic intraoperative image showing clips of venous vessels afferent to the internal iliac vein, and of the obturator vessels near to the ILNR.

**Figure 5:**
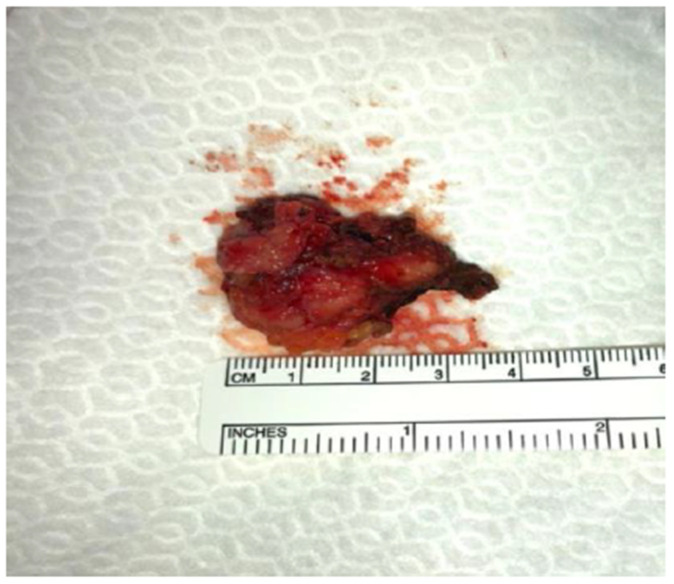
ILNR sample.

At laparoscopic exploration, severe pelvic visceral adhesions were found, with no signs of peritoneal carcinomatosis nor ascites. Following a careful adhesiolysis, the lateral and medial features of the right external iliac vessels were exposed by access to ileo-lumbar space. On the right side, the obturator nerve, the lumbo-sacral trunk and vessels were gradually and gently isolated (Figure 3). After closure by clips of venous vessels afferent to the internal iliac vein, and of the obturator vessels, the suspected node was removed (Figure 4 and Figure 5).

**Figure 6:**
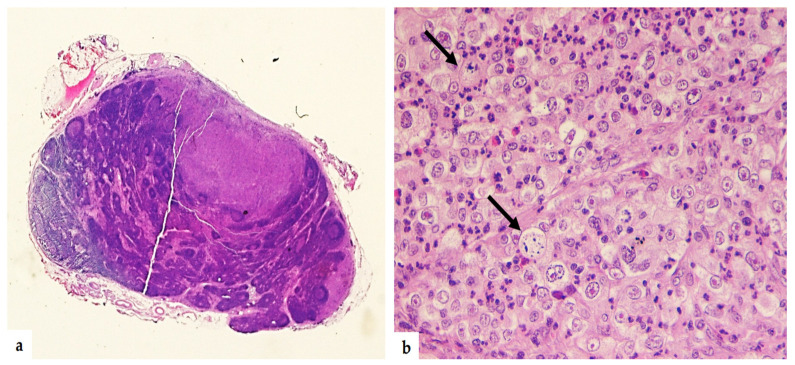
Isolated lymph node metastasis. Hematoxylin and Eosin image without magnification shows serous ovarian cancer intranodal recurrence (**a**). 40× Hematoxylin and Eosin shows cell clusters (arrows) within lymph node parenchyma with big nucleoli and significant nuclear atypia (**b**).

Straight-stick laparoscopy successfully managed the entire intervention, and no intra-operative complications occurred. The whole operating time was 125 min. The estimated blood loss was 90 mL. The post-operative course was uneventful, and the patient was discharged three days after the procedure. Histological examination confirmed the serous ovarian cancer intranodal recurrence (Figure 6). Since the nature of the recurrence (isolated lymph-nodal), the previous maintenance therapy with PARPi, and the absence of residual tumor, we decided to do quarterly follow-up; actually, after 16 months, no recurrence was detected.

## 3. Discussion

ILNR is actually considered as a rare entity in the context of EOC since it affects only 1–5% of relapsed EOC [16,17]. These ILNR has been described as a unique clinical disease entity, moreover, several studies underline a better prognosis in terms of PFS and OS compared to peritoneal or parenchymal forms of recurrence [20,23,24,25,26]. Furthermore, the idea that the lymph-node metastases in ovarian cancers are more chemoresistance has already been proposed by Morice et al. in an analysis of 105 patients [27] and confirmed by several studies [20,21,22,23,24,25,26,27]. The lower chemosensitivity of ILNRs can be justified by a lower percentage of cells in S-phase [18] and a greater CD3^+^ and CD8^+^ cell infiltration compared to extranodal relapse [20]. For these reasons, surgical treatment assumes a crucial role. The repeated surgery for ILNR, even in platinum-sensitive ovarian cancer, remains an important tool and this has been confirmed by several previous studies which highlighted the prognostic benefit in those patients [13,14,15,22,26,28]. Diagnostic laparoscopy also plays an important role in identifying a more diffuse disease and preventing unnecessary laparotomies delaying the chemotherapy [29]. Furthermore, in some cases, intraoperative laparoscopic ultrasound prevented conversion to laparotomy, guiding the surgeon in identifying lymph node recurrence in patients with anatomical alterations [30]. In literature, the prevalence of pelvic lymph node dissection managing pelvic ILNR ranges from 24 to 38%, and the surgery-related complication rate is meager [13,14,15,18,28,31]. Carefully selected patients with secondary platinum-free interval longer than 2 years or 2 years from secondary cytoreductive surgery, and an isolated recurrence, and prediction to achieve complete surgical resection, represent the ideal candidates for tertiary cytoreductive surgery, even if a higher risk of vascular lesion must be considered [9]. However tertiary cytoreductive surgery can be particularly complex and therefore requires surgical experience and skills that only a gynecologic oncology surgeon can reach [10]; also a specific training in minimally invasive surgery (either laparoscopy and/or robotic) is needed [10,32,33].

Moreover, the management of platinum-sensitive ROC, must consider *BRCA* 1 *or* 2 mutational status, as evidenced by Marchetti et al. [5] or in specific isolated forms of recurrence such as the hepatic one [21]. However, recently the ever-increasing number of patients who underwent further relapses after PARPi administration leads to the need for an evaluation of the prevalence of *BRCA* 1 *or* 2 gene mutational status on specific relapse settings, such as in the case of ILNR; even because treatment with a PARPi should not be offered to patients with recurrent EOC that have received a PARPi [34]. Even if recently, in the Phase IIIb OReO/ENGOT Ov-38 trial (NCT03106987), maintenance olaparib rechallenge significantly improved PFS in patients with platinum-sensitive ROC regardless of their *BRCA* status.

Hollis et al., reported a molecular characterization of ILNR that did not show marked enrichment or depletion of cases with BRCA mutational when compared to isolated extranodal relapses counterparts [20].

Gallotta et al., showed that *BRCA* 1 *or* 2 mutational status does not appear to be associated with better clinical significance in terms of OS and PFS in ILNR, differently from what happens in relapses in other sites [22]. This is in line with analysis by Delangle et al. that reported how ILNR in EOC does not seem to be associated with a better prognosis in terms of OS when compared to isolated peritoneal recurrence; but interestingly, the number of *BRCA* 1 *or* 2 mutated patients was statistically higher in the ILNR group [17].

In this context, the management of ILNR and the choice of a more personalized strategy be related to a series of factors: since the *BRCA* mutational status does not currently have an impact on ILNR, and the number of *BRCA* 1 *or* 2 mutated patients with ILNR who previously underwent PARP is set to increase, surgery in highly selected patients appears to play a crucial role.

In our specific case, we proposed minimally invasive tertiary cytoreductive surgery because at the time of the diagnosis the patient presented a pelvic ILNR recurrence, with a highly predictable optimal cytoreduction. Moreover, the patient had already undergone treatment with platinum and maintenance with PARPi in the previous peritoneal recurrence; therefore, in particular cases, surgery could adequately represent the possibility of achieving the best clinical benefit. We believe that the number of this kind of patients with specific isolated recurrence settings, already treated with PARPi, is destined to increase. For these reasons, these knowledges are precious to personalize the treatment in the early future. However, further prospective studies are needed to investigate the prevalence and the role of the *BRCA* 1 *or* 2 mutational status in the ILNR; in addition to the molecular biology of this rare disease, still little known today. Because of the lack of studies on this topic, our case report represents an isolated case and emerges from a choice of personalization of the treatment. Despite that, it is our opinion that our research can be a useful source for a deeper investigation to other expert center groups.

## 4. Conclusions

Our experience confirms that tertiary cytoreductive surgery can be considered an effective therapeutic option for the ILRN’s management even in patients *BRCA* 1 *or* 2 mutated already treated with PARPi. The personalization of the strategy and the achievement of a complete cytoreduction must be the aim of the treatment. To date, the impact of the *BRCA* 1 *or* 2 mutational status on this specific recurrence setting is still under investigation, therefore further studies will allow us to have more adequate knowledge in the management of increasingly complex patients.

## Data Availability

The authors confirm that the data supporting the findings of this study are available within the article.
